# Alzheimer’s-related brain damage shows sex-specific links with risk factors

**DOI:** 10.1371/journal.pbio.3001905

**Published:** 2022-12-14

**Authors:** Laurel A. Beckett

**Affiliations:** Department of Public Health Sciences, School of Medicine, University of California, Davis, California, United States of America

## Abstract

Alzheimer’s disease is marked by brain damage from tau and amyloid aggregates, especially to the hippocampus and its default network. This Primer explores a new study in PLOS Biology which reveals differences between men and women in the patterns of damage and in its association with risk and protective factors.

## Teaser

Alzheimer’s disease is marked by brain damage from tau and amyloid aggregates, particularly to the hippocampus and the default network. A new study in *PLOS Biology* shows sex differences in the patterns of damage and in their association with risk and protective factors.

Around the world, Alzheimer’s disease (AD) and related dementias (ADRD) affect an estimated 55 million people [1]. AD is a progressive dementia that affects memory, thought, and behavior through damage to the brain cells or neurons. The hallmarks of brain damage are tau protein deposits that build up inside the neurons and amyloid plaques that accumulate in the spaces between the neurons [2]. One of the first parts of the brain to be affected by AD damage is the hippocampus, the center of learning and memory in the brain [3].

While the causes of AD are not fully understood, genetics has a role. People who inherit one or two copies of the ε4 allele of the apolipoprotein E (APOE) gene are at greater risk than those with the more common ε3 allele, while those with one or two copies of the much less common ε2 allele are at lower risk. In general, people with a family history of dementia are also at greater risk. Additionally, about 5 women are affected for every 3 men [4]. In this issue of *PLOS Biology*, Savignac and colleagues report results from a very large population-based study, the UK Biobank, which looked beyond sex differences in incidence of AD to whether the disease pattern itself might differ [5]. They explored how those patterns were related to genetic, environmental, and lifestyle factors and how those relationships differed between men and women.

Previous work has often treated the hippocampus as a single, undifferentiated region. Savignac and colleagues theorized that patterns of damage might be more subtle than could be captured by just hippocampal volume [5]. They examined not only the hippocampus itself, but also the default network (DN), a system that helps integrate cognition within the brain. They assessed the volume of 38 hippocampus subregions and 91 DN subregions, then constructed 25 separate pairs of hippocampus–DN summaries or signatures that captured a complete and much finer-grained picture of the AD-related volumetric variation of the hippocampal region than total hippocampal volume alone. This novel approach showed considerable variation across people in localized patterns of brain volume and motivated a deeper look at the subregion signatures that showed the greatest variation from person to person.

What might account for this heterogeneity in AD-related brain volume? Might it be related to differences in health outcomes, and might sex differences have a role? The investigators examined the associations between brain–volume patterns and *APOE* genotype and, in turn, their relationship to phenotype characteristics including both outcomes and risk factors (cognitive and physiological assessments, physical and mental health assessments, sociodemographic characteristics, and lifestyle factors). Men and women were analyzed separately. The large number of models and variables considered required careful adjustment for multiple comparisons to avoid false discoveries, and internal cross-validation to validate the findings.

**Fig 1:**
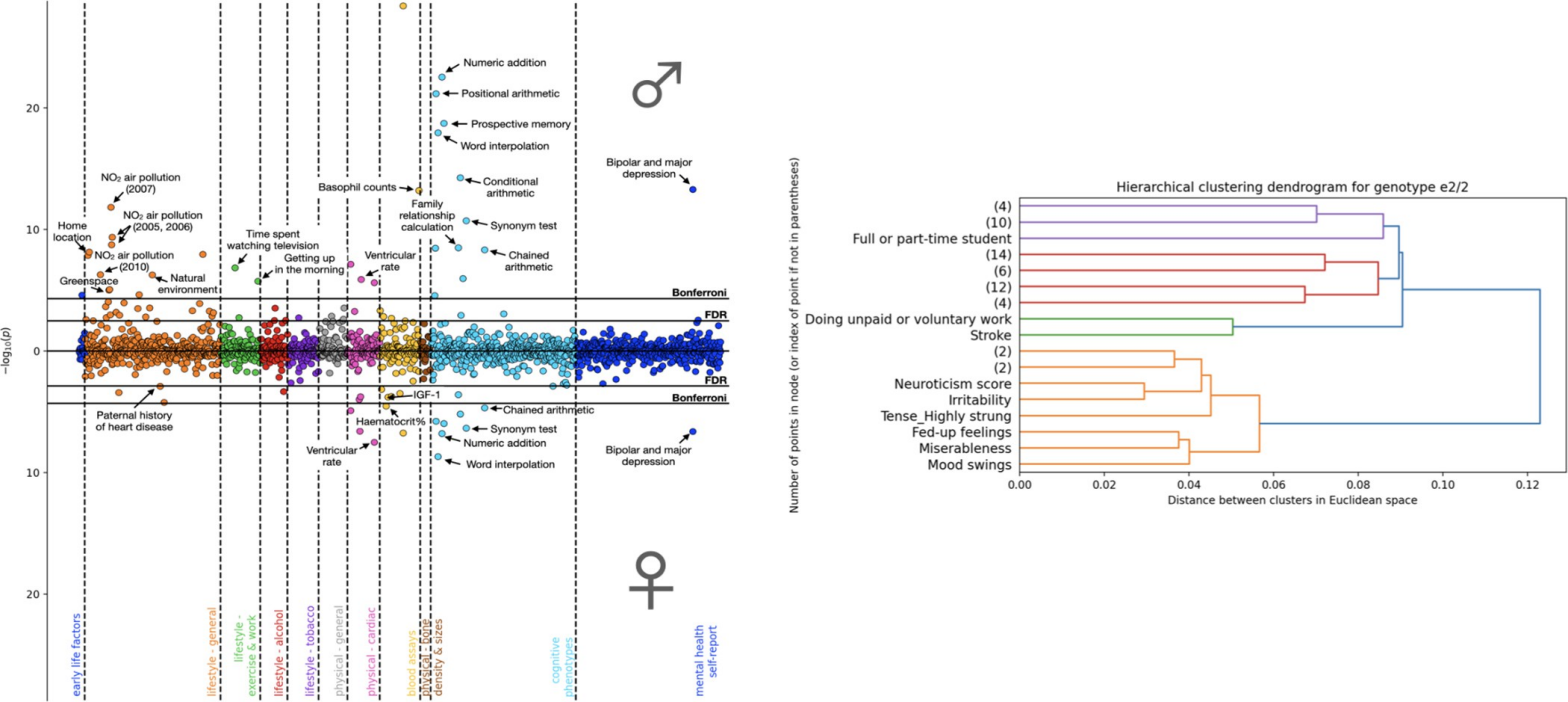
Relationship of risk factors to hippocampus–DN signatures is both sex specific and *APOE* genotype specific. Savignac and colleagues [5] created 25 summaries of hippocampus and default network (DN) subregion brain volume measures and then ranked by how strongly each subregion summary was associated with genetic risk conferred by the individual’s *APOE* genotype. The left-hand diagram illustrates the correlations between brain volume summaries in the region most reflective of genetic risk and phenotypic measures, separately for men (top dots) and women (bottom dots). Cognitive measures (turquoise dots, second to right block) and depression (dark blue, right block) were associated with brain volume for both sexes, as were cardiovascular risk factors (magenta dots). Air pollution measures were associated only for males (orange dots, second to left). This shows a sex-specific pattern of association between risk factors and volumetrics for hippocampal subregions most strongly associated with genetic risk. The investigators also examined the correlation of risk factors with hippocampus–DN signatures separately for each of the six *APOE* genotypes and grouped risk factors into clusters with similar patterns of association. The right-hand diagram shows risk-factor groupings for the ε2/2 genotype. The orange cluster at the bottom of the diagram shows that similar brain volume patterns occur within this genotype for traits typically associated with neuroticism such as irritability, miserableness, and mood swings. This suggests that the overall protective effect of the ε2/2 genotype may be modified by personality traits.

The *APOE* genotype was represented by a 5-point risk scale from −2 to +2, calculated as the number of ε4 or risky alleles a person had minus the number of ε2 or protective alleles (ε3 alleles were treated as neutral) [5]. Some localized volumetric patterns were more strongly associated with high genotypic risk, suggesting that risk is targeted (and protection conferred) to specific key areas of the hippocampus and its related DN. The localized volumetric pattern measures were then examined as predictors of phenotype characteristics. Unsurprisingly, in both sexes, higher levels of cognitive impairment measures were the most frequent phenotypic variables correlated with higher levels of genotype-linked brain volume differences (Fig 1). In addition, in both sexes, higher levels of *APOE*-linked brain variation were associated with depression and some cardiovascular disease markers. Men showed stronger associations with environmental factors, including air pollution, and with verbal–numerical reasoning. Women showed additional associations with cardiovascular phenotypes beyond those reported for men.

The investigators then explored whether phenotype might relate to brain volume patterns differently within the six *APOE* genotypes (*APOE* ε2/2, ε2/3, ε2/4, ε3/3, ε3/4, ε4/4.) The most striking finding was that personality traits associated with neuroticism showed a distinct pattern of association with hippocampus–DN covariation expressions in ε2 homozygotes (Fig 1). This suggests that protection conferred by ε2 for AD-related brain damage might be modified by these traits. Another key finding in ε2 homozygotes was that social lifestyle factors in men and physical activity factors in women were associated with lower levels of brain damage. A related analysis found sex specificity in brain volume–*APOE* interactions, with a suggestion that ε2 can still be somewhat protective against ADRD in women, even in the presence of an ε4 allele.

The study by Savignac and colleagues [5] adds to the previous literature an anatomically detailed look at the pattern of brain volumes in subregions of the hippocampus and the DN. Importantly, the investigators showed substantial person-to-person variation in these patterns and that specific regional differences are related to genetic risk/protection and to cognitive outcomes. This suggests that future work with MRI data should consider more fine-grained summaries of hippocampal atrophy. Focus on finer regions gave insight into sex-specific differences in associations with phenotype, particularly environmental risk (men) and added cardiovascular risk (women). Finally, the large sample size permitted an in-depth analysis of the effects of the *APOE* ε2 allele, including the novel finding that it may be protective even in the presence of an ε4 allele, at least in women.

Strengths include the use of a large population-based data resource and careful high-level multivariate analysis. Generalizability may be limited, however, by the UK setting. Some studies have reported that the impact of *APOE* genotype differs across racial groups, with black individuals having less added risk from ε4 [6], while others have suggested that education may account for a sizable proportion of racial disparities in risk [7]. Future research is needed in other, more diverse population-based settings. Research is also needed to examine further the degree that the role of modifiable risk factors like social lifestyle and physical activity is both sex specific and genotype specific and that these might contribute to sex differences in AD incidence.

## Abbreviations

AD: Alzheimer’s disease
APOE: apolipoprotein E
ADRD: Alzheimer’s disease and related dementias
DN: default network
